# How to Catch Customers’ Attention? A Study on the Effectiveness of Brand Social Media Strategies in Digital Customer Engagement

**DOI:** 10.3389/fpsyg.2021.800766

**Published:** 2021-12-15

**Authors:** Yuying Liu, Xinxin Liu, Meng Wang, Decheng Wen

**Affiliations:** School of Management, Shandong University, Jinan, China

**Keywords:** digital customer engagement, brand social media strategies, content strategy, response strategy, brand image, discretionary purchases

## Abstract

Enterprises often post branded content on social media and adopt a proactive response approach to improve digital customer engagement to gain a competitive advantage. However, there are many brands which fail to operate social media as effectively as expected. The effective use of brand social media strategies to improve digital customer engagement remains an ongoing challenge for the enterprises. Based on firm-generated content theory and social presence theory, this study aims to identify the impact of brand social media strategies on different levels of digital customer engagement, including positive filtering, cognitive and affective processing as well as advocacy from content strategy and response strategy. Based on 1,519 brand posts on the official Weibo pages of eight of the top 500 Chinese brands in 2021, this study uses a multiple linear regression model to examine the impact of brand social media strategies on digital customer engagement and the moderating effects of brand image and discretionary purchases. The findings show that, on the one hand, among the brand social media content strategies, action content strategy is associated with higher levels of digital customer engagement. On the other hand, different brand social media response strategies have a differential impact on digital customer engagement levels, with cohesive response being the best strategy for increasing digital customer engagement level. In addition, the effectiveness of brand social media response strategy in digital customer engagement is further moderated by the brand image and discretionary purchases. In contrast, the effectiveness of brand social media response strategy in digital customer engagement is stronger when the brand image emphasizes its “competence” or the discretionary purchases focus on “material purchases.” This study not only enriches the research on digital customer engagement but also provides a reference for the brand strategy selection, design and management based on social media.

## Introduction

In today’s highly connected network environment, the common use of social media is changing the environment for branding. The interactive, participatory, and open natures of social media offer brands the opportunity to connect and create online content, more and more brands are noticing the importance of social media when it comes to areas such as exploring consumer needs and building online reputation, and are therefore striving to provide engaging branded content as well as adopting an responsive approach to consumers to gain a competitive advantage, which can be shown through digital customer engagement (Jansen et al., 2009; Cespedes, 2015). Eigenraam et al. (2018) suggests that digital customer engagement are consumers’ online behavioral manifestations of brand engagement that go beyond purchases. Consumer social media practices such as browsing, liking, sharing, commenting can be conceptualized as behavioral manifestations of customer brand engagement. Digital customer engagement is growing in importance as a source of brand value, as it increases consumer loyalty and affects brand performance. Recent research has shown that digital customer engagement is a key performance metric for evaluating a brand’s social media strategy and has therefore attracted much attention in various research areas (McShane et al., 2021; Shahbaznezhad et al., 2021). However, many enterprises find it a difficult task to successfully deploy social media strategies to develop customer engagement, trust and relationship (Maclnnis et al., 1991; Effing and Spil, 2016). Therefore, enhanced insights into the effectiveness of brand social media strategies for digital customer engagement contribute to our understanding of this critical area. Furthermore, different brand images and type of discretionary purchases may influence digital customer engagement, as brand images are not only associated with media images, but also cover various types of content related to the business, which can influence consumers’ brand perceptions or associations. Accordingly, the distinction between discretionary purchases can indirectly affect consumers’ brand-related cognitive, emotional and behavioral activity. However, academic understanding in this area is still relatively scarce, thus creating an important knowledge gap.

In this study, we mainly solve the following questions: (1) Which type of brand social media content strategies are more likely to influence digital customer engagement? (2) Do all brand social media response strategies affect digital customer engagement? (3) How do different brand images and types of discretionary purchases affect digital customer engagement? Answers to these questions will help to understand brand social media strategies, including which strategies are likely to have the greatest impact on digital customer engagement, and which brand sources and components they should pay particular attention to when predicting the impact on digital customer engagement. To answer the above key research questions, this study explores the impact of brand social media strategies on digital customer engagement in the following ways: (1) Based on customer engagement theory, this study conducts a comprehensive study of digital customer engagement from hierarchical differences and overall level. (2) Based on the firm-generated content theory and social presence theory, we focus on investigating the impact of two key brand social media strategies on digital customer engagement, which are content strategy and response strategy, and determine which brand social media strategy is more effective by comparing the level of digital customer engagement. (3) From a comparative perspective, the level of digital customer engagement varies significantly by brand image and discretionary purchases. Therefore, this study focuses on the moderating effect of brand image and discretionary purchases.

## Literature Review and Research Hypotheses

### Digital Customer Engagement

According to the existing literature, customer engagement is a complex concept with cognitive, emotional and behavioral dimensions (Voorveld et al., 2018; Hollebeek, 2019). Brodie et al. (2011) define customer engagement as “a psychological state that arises from diverse experiences.” Subsequently, Cheung et al. (2021) conceptualize customer engagement as “the voluntary engagement of customers in interactions with brand,” centering on specific levels of cognitive, emotional, and behavioral dimensions that occur in consumer-brand interactions. In addition, customer engagement reflects the motivational state of the consumer and appears as “motivated non-transactional behavior” (van Doorn et al., 2010). Chen et al. (2019) and Chen et al. (2020) distinguish two CE orientations—rational and emotional CE, which lead to different behavior patterns, and indicate firms in the online market especially strive to increase customer engagement. Customer engagement is built on the development of digital technologies and social media, and its characteristics have changed subtly. What we focus on is how consumers perform in the online environment, i.e., digital customer engagement. The main features of digital customer engagement are specific online contexts, conversational interactions, and exchanges that go beyond the purchases or consumption of products or services. Mollen and Wilson (2010) describe online engagement as “a cognitive and affective commitment to an active relationship with the brand as personified by the website or other computer-mediated entities designed to communicate brand value. It is characterized by the dimensions of dynamic and sustained cognitive processing and the satisfying of instrumental value (utility and relevance) and experiential value (emotional congruence with the narrative schema encountered in computer-mediated entities).” Gavilanes et al. (2018) considers digital customer engagement as the interaction between consumers and brands in the online environment. Hollebeek and Macky (2019) define digital content marketing’s engagement as the dynamic process of brand engagement, namely cognitive engagement (i.e., a consumer’s level of brand-related thought processing and elaboration), emotional engagement (i.e., a consumer’s level of positive brand-related affect in the interactive process of digital content marketing), and behavioral engagement (i.e., a consumer’s level of energy, effort and time consumers spend on using a brand). Eigenraam et al. (2018) classifies digital customer engagement practices into five distinct types based on a taxonomy of consumers, namely, fun practices, learning practices, customer feedback, work for a brand, and talk about a brand. The digital customer engagement research is increasingly segmented. Given these definitions, this study defines digital customer engagement as “the cumulative process by which consumers engage in diverse voluntary inputs to a brand in an online environment, which goes beyond the core transaction to build or maintain a long-term stable brand relationship.”

Regarding the operationalized aspects of digital customer engagement, digital customer engagement is regarded as a continuum of brand-related activities from high to low, exhibiting different levels (Muntinga et al., 2011; Schivinski et al., 2016). Existing research confirms the impact of brand observable social media behaviors on digital customer engagement and that metrics such as likes, comments and shares can represent different levels of engagement (Ji et al., 2019; McShane et al., 2021; Shahbaznezhad et al., 2021; Unnava and Aravindakshan, 2021). For example, Thompson and Brouthers (2021) divide digital customer engagement into share engagement behavior and click engagement behavior, arguing that the two have completely different characteristics. Share engagement behavior refers to advertising brand, product or service information through the sharing options provided by digital platforms; whereas click engagement behavior is where focal consumers measure corporate-generated content to improve their experience with a specific product or brand (van Doorn et al., 2010; Thompson and Brouthers, 2021). Dhaoui and Webster (2021) divide customer engagement behavior on social media into five constructs, namely endorsement, feedback, recommendation, conversation, and consensus; while Kim and Yang (2017) and Srivastava et al. (2018) point out that each engagement behavior differs in value and commitment of resources. Like-level engagement is the simplest level. The digital value of comment-level engagement is higher than like, owing to comment is a behavior of interactive relationship and is related to the specific rhetorical context. Sharing-level engagement related to self-presentation as the highest level. Synthesizing approaches widely used by several scholars in the field, we consider it as a continuum of high and low levels, constructing digital customer engagement as a three-level structure on the DCE model proposed by Gavilanes et al. (2018), with (1) positive filtering: reactions to content showing positive emotional states (likes); (2) cognitive and affective processing: collaborative creation of content in the brand environment (comments); and (3) advocacy: stronger cognitive and emotional investment, value co-creation, publishing, and self-expression (shares).

### Brand Social Media Strategies

Social media strategies have evolved based on web 2.0 thinking and digital technologies (Li et al., 2021). Several category frameworks exist to classify social media strategies, all involving dimensions such as content arrangement, conversation (Kaplan and Haenlein, 2010; Kietzmann et al., 2011). In subsequent work, Effing and Spil (2016) defines social media strategy as “a goal-directed planning process for creating user generated content, driven by a group of Internet applications, to create a unique and valuable competitive position.” The key elements of a social media strategy include target audience, channel selection, goals, resources, policies, monitoring, and content activities (Effing and Spil, 2016). In light of the above research, we define brand social media strategy as “the process of developing and executing a set of activities by which a brand uses social resources to create distinct brand value.” Brand content and brand response are core components of brand social media strategy (Men et al., 2018). Therefore, we understand the effectiveness of brand social media strategy from the dimensions of content strategy and response strategy, based on the comparison of three content categories and three response categories.

#### Brand Social Media Content Strategies

According to Firm-Generated Content theory, firm-generated content (FGC) is a multidimensional construct. In terms of content characteristics, firm-generated content are divided into informative and persuasive content, which mainly reflect entertainment value and information value (Demmers et al., 2020). Marketers divide social media content into information, entertainment, social and remuneration content, and argue that user interaction (e.g., likes, shares) varies according to the type of content (Chandrasekaran et al., 2019). We adapt the definition of firm-generated content by Kumar et al. (2016) to the context of our study. We define brand social media content strategy as “brand-related content created by a brand on its official social media page, but also any cue that is intentionally designed to convey the brand’s tone of voice and personality” (Kumar et al., 2016; Colicev et al., 2019).

Lovejoy and Saxton (2012) proposed the ICA framework applying to social media functions: Information, Community and Action, which is also the mainstream social media content classification framework at present. In this paper, we combine the relevant content features of FGC and choose ICA framework as the brand social media content strategy classification. (1) Information, which mainly contains content that conveys brand-related information, focused events or news, facts, reports or industry knowledge relevant to the organization’s stakeholders. This strategy favors a simple one-way exchange of information; (2) Community, which encourages stakeholder interaction, sharing and conversation. Community involves two main categories, acknowledgment of current and local events (e.g., holiday greetings) and recognition and appreciation to the consumers, which are primarily related to the “community building” element; “responding to public messages” and “collecting responses” are more directly related to “conversation”; (3) Action, which consists mainly of consumer appeals for product promotion, event promotion, and participatory promotions, such as contests, quizzes or competitions, rewards, and asking followers to do something specific to help the firm achieve its goals (Lovejoy and Saxton, 2012).

Previous research has shown that the amount and category of brand content published have an influence or interaction on customer engagement (Su et al., 2015; Lee et al., 2018; Barreto and Ramalho, 2019; Meire et al., 2019; Labrecque et al., 2020). The extent to which brand social media content strategies affect digital customer engagement varies. First of all, not all content strategies stimulate the same level of customer engagement (de Vries et al., 2012; Dhaoui and Webster, 2021; Liu et al., 2021; Shahbaznezhad et al., 2021). Chandrasekaran et al. (2019) state that the level of customer engagement varies by content category, with remuneration and social content category showing significant differential effects in the number of likes and shares. In the research by Dolan et al. (2019), informational content positively influenced the number of likes and shares, however, there was no evidence that informational content influenced the comments, while neither entertainment nor relational content, in the form of comment or share, demonstrated a relationship with digital customer engagement. Therefore, we assume that the different levels of digital customer engagement vary by content type.

*H1: Community (vs. Information) has stronger effects on positive filtering (a), cognitive and affective processing (b) and advocacy (c).*

*H2: Action (vs. Information) has stronger effects on positive filtering (a), cognitive and affective processing (b) and advocacy (c).*

#### Brand Social Media Response Strategies

Based on social presence theory, mediated communication is social in nature (Yue et al., 2019). Social presence is defined as “the degree of salience of the other person in the communication and the consequent salience of the interpersonal relationships” (Cui et al., 2013, p. 662). Short et al. (1976) put forward “the significance of the other when using communication medium of social presence and the significance of the resulting interpersonal interaction” from the perspectives of media. Social presence captures the interpersonal nature and relational orientation of communication. Social presence is an important perception in the media environment that provides a way to enhance communication or interaction between enterprises and consumers (Tu, 2000; Osei-Frimpong and McLean, 2018). Social presence theory considers advertisers presenting brands as “real” and positions them as the “contacts” in relation to consumers by actively and strategically enhancing social presence to facilitate online interaction and conversation (Men et al., 2018). With the brands being the messengers, response strategies can be defined as “the communication process by which brands generate non-verbal and verbal cues in order to enhance intimacy with (potential) consumers.” The social presence dimension involving interpersonal relationships is also particularly relevant to the study of brand information (Osei-Frimpong and McLean, 2018; Karampela et al., 2020).

The three response strategies of social presence involve non-verbal and verbal cues generated or created by enterprises on social media. Response strategies consist of three categories: (1)Affective response identified with emojis, humor, and self-expression as key features that facilitate social interactions; (2)Interactive response involving asking questions, agreeing, showing appreciation, or supporting others in ways that help form a personalized impression of the interlocutor; (3)Cohesive response, which serves to maintain or build a sense of community or group commitment, reduce social distance, and enhance brand consensus. Previous research argues that social presence is related to consumer engagement (Fortin and Dholakia, 2005; Tang and Du, 2021). For Algharabat et al. (2018), social presence positively impact consumer brand engagement. Men et al. (2018) and Yue et al. (2019) further identify response strategies as important factors that may facilitate or influence digital customer engagement and as the basis for explaining the relationship between brand social media response strategies and digital customer engagement. Yue et al. (2019) found that three response strategies have differential effects on different levels of consumer behavior. Among them, affective strategy is positively related to the number of likes and comments and negatively related to the number of sharing. To gain insight into the impact of brand social media response strategies on digital customer engagement, we take a brand behavior perspective, instead of a consumer perspective, focusing on how companies construct conversations, which differs from the existing information literature. We apply the framework proposed by Rourke et al. (1999), using voluntary disclosures in place of the self-disclosure dimension of affective response to better reflect the characteristics that define them in this study (Zhang and Lin, 2015). Voluntary brand disclosure, which includes a firm’s ability to provide useful information that is attractive to stakeholders, transparency and openness are key aspects of disclosure (Abitbol and Lee, 2017). Information posted by enterprises on their social media pages, e.g., financial transaction disclosures, management decisions such as job appointments, social responsibility disclosures, environmental disclosures and brand crisis disclosures, may enhance stakeholders’ sense of belonging. Given the above research, the level of digital customer engagement will also vary depending on the categories of response strategies. Therefore, we make the following hypotheses:

*H3: Affective response positively influences positive filtering (a), cognitive and affective processing (b) and advocacy (c).*

*H4: Interactive response positively influences positive filtering (a), cognitive and affective processing (b) and advocacy (c).*

*H5: Cohesive response positively influences positive filtering (a), cognitive and affective processing (b) and advocacy (c).*

### Brand Image

In the field of branding research, Keller defines brand image from the Consumer-Brand Perception Framework as “the perception of a brand as reflected by the brand associations in the consumer’s mind, which are those information points in the consumer’s mind that are connected to the brand’s information points and that contain the brand meaning to the consumer (Keller, 1993).” Focusing on the social media context, the stereotype content model is interpreted as an appropriate element of brand image when brands reflect their interpersonal or social relationships and actively construct consumer brand relationships through social media. The mixed stereotype content model proposed by Fiske et al. (2002) assumes qualitative differences in stereotypes and prejudices of different groups, while arguing that how people perceive social groups are distinguished by two main dimensions, namely “warmth” and “competence.” “Warmth” is defined as the perceived intentions of the social group / individual, and “competence” is the capability to pursue it. Various studies have tested and validated stereotype content model, applying them to a variety of social goals or social objects, e.g., from individual perceptions to national perceptions (Cuddy et al., 2004, 2009), and have shown that the two core dimensions (competence and warmth) guide people’s decisions and interactions with other people and social groups. Kervyn et al. (2012) applied the stereotype content model to brand perceptions, where different perceptions of brands would be valuable predictors of how consumers behave toward different brands. On the one hand, when presenting both abstract and concrete information about a brand, “warmth” focuses on expressing the brand message at a more abstract level than “competence,” meaning that the “competence” brand image is likely to drive people to look more at the performance characteristics of the brand, and consumers influenced by “warmth” will focus on information about the overall brand benefits, such as brand love or brand passion. Therefore, based on the mature social perception approach: the stereotype content model, the brand image framework is jointly interpreted in terms of two fundamental dimensions: “competence” and “warmth.” “Competence” can be seen as an assessment of the “functionality” of a brand, related to objective benefits such as efficiency or reliability, as well as to the inherent characteristics of brand attributes (such as price, design, and quality), which are defined as “competence.” Brand image is defined as “warmth” when the brand acts as a relationship builder, highlighting the symbolic benefits, quasi-human and self-expressive values of the brand, and attracting (potential) consumers in non-traditional ways or through quasi-social interactions. Therefore, we expect the impact on digital customer engagement to vary across image frameworks, with the effectiveness of warmth (compared to competence) expected to be more prominent on digital customer engagement.

*H6: “Warmth” image (vs. “Competence” image) has stronger effect on positive filtering (a), cognitive and affective processing (b) and advocacy (c).*

From the above discussion, even though brand social media response strategies may have some impact on digital customer engagement, this process may be moderated by brand image. The level of brand responsiveness in the “warmth” image framework is likely to be higher than that of “competence,” as it conveys and presents a need to expect social recognition and acceptance, with a primary focus on the “voice of the customer” to bring up brand preference of consumer (Kolbl et al., 2020). “Competence,” on the other hand, places more emphasis on the specialization of a product or service or the ability to highlight the strength of a brand in order to reinforce mutually beneficial values, for example, the company’s global success or industry leadership, the implementation of the company’s quality control program. As a result, “competence” is more informing in style, with fewer communication skills and lower levels of responsiveness. Previous literature has not identified how various types of brand image adapt on social media to promote digital customer engagement. We expect that brand social media response strategies that effectively drive digital customer engagement may vary by brand image, with “warmth” more likely to result in higher levels of digital customer engagement from brand social media response strategy.

*H7: Brand image (warmth vs. competence) has a moderating effect on the relationship between brand social media response strategy and digital customer engagement.*

### Discretionary Purchases

Distinguishing from research perspectives that focus on product categories and functional attributes, this paper frames the types of discretionary purchases as experiential purchases and material purchases. The main purpose of experiential purchases is the acquisition of a life experience: an event or a series of events, a person’s life experiences; material purchases refers to purchases made with the main purpose of acquiring material goods: tangible items that one can own (Van Boven and Gilovich, 2003). Several empirical studies have successfully demonstrated the differential performance of these two classifications of discretionary purchases in predicting the outcomes of various types of social and psychological experiments. For example, people derive more pleasure or happiness from freely chosen experiential purchases than from freely chosen material purchases (Kumar and Gilovich, 2015; Weingarten and Goodman, 2021). Furthermore, people tend to be more willing to share socially about experience-based consumption than material-based consumption, a difference that stems from users’ perceived social approval of the purchase (Zhang et al., 2021). Current research has extended to a new area primarily relevant to business, namely consumer effort, suggesting that consumers are more willing to make an effort to obtain experience-based consumption rather than material-based consumption. Additional views suggest that experiences offer consumers greater conversational potential than objects, and are closer to the self and more symbolic and socially significant than material things, considering over 70% of the daily conversations and posts on social media are about the self (Bastos, 2020). Following this logic, the strategies adopted by brands on social media are seen as unique ways to connect experiential or material types of purchases. Discretionary purchases also reflect the consumer’s purchase intention which in turn affects the strength of customer engagement for the brand. One of the main views of digital customer engagement is that “the participant conveys his/her personal perceptions or preferences to the brand or to others.” Compared with material purchases, experiential purchases seems to be more conducive to digital customer engagement, as experiences are more unique than material. Thus, discretionary purchases is a key factor in conveying the brand messaging. After comprehensive consideration, these theoretical frameworks and findings do support the prediction that consumers generate more digital customer engagement and have a positive impact on experiential purchases, and that the greater exchanging potential between experiential and material purchases would explain this effect. Therefore, this study makes the following hypotheses:

*H8: Experiential purchases (vs. Material purchases) has stronger effects on positive filtering (a), cognitive and affective processing (b) and advocacy (c).*

*H9: Discretionary purchases (Experiential purchases vs. Material purchases) have a moderating effect in the relationship between brand social media response strategy and digital customer engagement.*

## Materials and Methods

### Research Background

In recent years, many scholars (Pezzuti et al., 2021; Sheng et al., 2021; Unnava and Aravindakshan, 2021) have generally acknowledged the key role of social media in brand development and emphasized the positive impact of brand engagement behaviors on digital customer engagement. We collected a dataset from the social media platform Weibo. First of all, Weibo is the most popular open social media platform in China, with 511 million monthly active users according to the *Weibo 2020 User Development Report*, maintaining an obvious advantage over other platforms. Secondly, Weibo users can disclose personal or brand information such as interests, complaints, brand preferences, and brand-related experiences through text, images, and videos. Lastly, most of the social network studies used in research are based on or include Weibo data. Thus, Weibo becomes the most relevant social network for consumers and brands.

In order to emphasize brand generalization and comparison, this paper selects *Brand Finance*’s list of Top 500 Brands in China 2021 as a brand selection criteria to further take in effective social media strategies. This sampling framework has several advantages over lists on other business lists such as *Fortune*. First, since other lists cover brands on a global scale, cross-cultural factors such as national sentiment or cultural differences might lead to additional influence, therefore, choosing this list is more appropriate for this study to investigate from the same cultural context. Second, the top-ranked companies on the list are involved with multiple industries, including technology, media culture, airlines, and restaurant business, which may bias the findings toward two discretionary purchases types. Third, most of the brands on the list post relevant information on Weibo, which makes Weibo a practically important approach to get to the consumers.

### Sampling and Data Collection

#### Brand Selection

Based on *Brand Finance*’s assessment, this study selected the brands by following the criteria below. First, to prevent industry homogeneity, multiple categories of industry representatives were selected. Second, it has been observed that many brands have set up more parallel accounts based on sub-brand or product categories. In order to maintain consistency and comparability with other brands with unique accounts, we chose to collect data about the company’s brand level. Lastly, brands with less than 6 months of Weibo postings and low activity are filtered out.

Before the formal survey, discretionary purchases were investigated for brand screening using an online questionnaire, and a total of 89 online questionnaires were collected. In the pretest, information on several real companies from 18 different industries (e.g., media culture, apparel, cosmetics, banking, airlines, technology, communications, etc.) were provided. Building on the research of Van Boven and Gilovich (2003) and van Doorn et al. (2010), respondents were asked to think about their purchases experience and to choose a type of discretionary purchases within the industry to maximize this difference (e.g., a plane ticket vs. a refrigerator). They determine the type of purchases on industry attributes by choosing options like “experiential purchases,” “material purchases,” “unsure” or “refused to answer,” to indicate their perceptions of both experiential and material purchases categories. Based on the survey results, a total of four Chinese brands with predominantly experiential purchases were selected: Air China, China Southern Airlines, Tiktok, and Tencent; and the four Chinese brands with predominantly material purchases: SAIC Motor, Geely Auto, Xiaomi, and Huawei. These eight companies have high brand familiarity and a relatively comparable number of brand followers in 2021, and post a similar amount of content on Weibo during 2020 and 2021.

#### Data Collection

The time span for data collection in this study was from June 1, 2021 to August 31, 2021 (Chandrasekaran et al., 2019). Firstly, this time span increases the longitudinal nature of the dataset, which helps to further reduce the prevalence problem. Secondly, it allows us to mitigate additional effects due to transient activity events. We use python to collect numerical customer engagement related metrics for all posts and their corresponding likes, shares, and comments during the time span. After data cleaning, the final dataset contains 1,519 social media posts, 40,142 comments, etc. Due to the default settings of Weibo and the limitations of the data crawler, we only collected about 70–80% of the top-ranked comments.

### Operationalization of Variables

In order to capture the digital engagement between the company and its current users, we collected data about the company and user activities and interactions on the official brand Weibo page. Our variables were divided into two categories: (i) brand-centric and (ii) user-centric. The brand-centric variables are independent and moderating variables in the study’s conceptual model to capture the brand’s ongoing efforts on social media. On the other hand, user-centric variables (dependent variables) show the extent to how users respond based on the brand’s social media strategy, i.e., digital customer engagement.

#### Independent Variables

##### Brand social media content strategies

Based on the literature and the theoretical framework presented in the previous section, we operationalized and tested three content strategy categories by measuring brand social media content strategies as categorical variables, namely 1 = “Information strategy”; 2 = “Community strategy”; and 3 = “Action strategy.” Two trained coders coded each blog post of the brands. If a Weibo post of a certain strategy type existed, it was coded as 1, while posts with no such content was marked as 0.

##### Brand social media response strategies

This study is carried out on the research basis of Kent and Taylor (1998) and Rourke et al. (1999) to explore the extent to which brand social media response strategies reflect the brand social presence. It is also based on three communicative strategies and related metrics developed by Rourke et al. (1999) that contribute to social presence. Specifically, affective response can be expressed on social media through (i) emoji; (ii) humor; and (iii) voluntary disclosure.

Interactive response refers to explicitly identifying other communication partners’ messages by (i) continuing the conversation by explicitly referencing other messages, (ii) asking questions, (iii) expressing appreciation/compliment, and (iv) expressing consent.

Cohesive response uses (i) name to address people or reference to members of the public (both internal and external); (ii) inclusive pronouns to address or refer to groups (e.g., we, community, society); and (iii) purely social features, greetings, and other social techniques to maintain brand sentiment.

##### Brand image

Brand image is a dummy variable divided into “warmth” and “competence” (0 = competence, 1 = warmth).

##### Discretionary purchases

To determine whether there is a purchases type difference in the impact of brand social media response strategy on digital customer engagement, discretionary purchases is set as a dummy variable divided into experiential purchases and material purchases (0 = material purchases, 1 = experiential purchases).

#### Dependent Variables

Positive filtering, cognitive and affective processing, and advocacy represent the different levels of digital customer engagement. The number of likes, comments and shares is calculated by counting user activity per post.

##### Positive filtering

“Likes” is the most common engagement practice on Weibo. According to the above definition, “like” is a form of positive evaluation of branded content (Gavilanes et al., 2018). Therefore, we describe hitting the “like” button as a weak form of DCE but showing a positive emotional state toward the brand or the content.

##### Cognitive and affective processing

We measured the number of consumer comments as a primary metric of cognitive and affective processing. Consumer comment is a good metric of the level of digital customer engagement. On Weibo, the number of comments represents the opportunity for consumers to see brand posts, reflecting their level of cognitive and affective processing (van Doorn et al., 2010).

##### Advocacy

“Shares” plays an active role in spreading branded content and serves as a reliable source and the most powerful form of DCE (de Vries et al., 2017). Users tend to identify themselves, develop social relationships and influence others by providing information about the brand in their social networks and sharing content with others and different social groups. Therefore, we counted the number of shares to measure the advocacy dimension.

#### Control Variables

Next we examine the control variables in the model. We include these variables below to control for heterogeneity between brands, which could explain some of the observed differences in the impact of brand and digital customer engagement associations (de Vries et al., 2012; McShane et al., 2021).

##### Time control variables

First, **Weekdays**. Weekend post has a significant impact on enterprise content strategy, and we use it to capture weekend and weekday seasonality (Hughes et al., 2019). Weekdays is a metric variable to determine whether posts occur on weekends (=1) or weekdays (=0). Second, the **posting schedule**. Posting schedule is considered as a key consideration for account managers when designing social media strategies, and the timing of posting predicts customer engagement (Sabate et al., 2014; Shahbaznezhad et al., 2021). Therefore, our posting schedule variables were dummy coded to divide the time into two parts: posts from 8 a.m. to noon and 2 p.m. to 5:30 p.m. (working hours) were marked as 1, and posts from noon to 2 p.m. and 5:30 p.m. to 8 a.m. (resting time) were marked as 0.

##### Brand control variables

The level of digital customer engagement may vary with the number of brand followers vs. the level of brand activity. Given the study by Dhaoui and Webster (2021), this paper adds three variables, which are the number of followers, overall posting volume and posting frequency, to observe the extent of their differences on digital customer engagement.

##### Media richness

Media formats include the corresponding ability to deliver messages containing rich information. This paper takes into consideration the objective characteristics of media formats that not only determine their ability to spread information but also trigger different levels of digital customer engagement. Building on the research by Tseng et al. (2017) and Shahbaznezhad et al. (2021) and others, media richness was operationalized as a categorical variable consisting of four dimensions:(i) topics; (ii) content and links; (iii) photos and images; and (iv) videos.

##### Length of content

Previous research has found that message length may positively or negatively affect outcome metrics. Therefore, we controlled the length of the content of the blog posts.

### Coding Procedures

To avoid subjective bias, the eight selected companies were randomly divided into two groups to be handled by two coders. Both coders received detailed training on the coding tool and classification set, and the coding process took approximately 2 weeks. The independent variable metrics were all coded according to a dichotomy of information presence (i.e., 1 or 0) to minimize possible subjective decisions by the coders (Kim, 2011). Inter-coder reliability was then calculated. We measured Cohen’s Kappa value (Cohen, 1960). The value of Cohen’s Kappa for brand social media content strategy is 0.797, the value of Cohen’s Kappa for brand social media response strategy is 0.846, and the value of Kappa for brand image is 0.855, suggesting “substantial agreement” between the coders (Viera and Garrett, 2005). In addition, some of the Weibo content in the extended dataset involving new message types were re-evaluated, requiring only the adjustment of existing subcategories rather than the construction of new subcategories (Coursaris et al., 2016; Gavilanes et al., 2018).

### Basic Model

In order to test the hypotheses, we used a multiple linear regression model with categorical variables (Long, 1997). To explain positive skewness, our first step is to add 1 for log-non-linear transformation by calculating the natural logarithm of likes, comments, and shares to avoid the possibility of taking log 0 as the dependent variable in the analysis (Ba and Pavlou, 2002). That is,


$${\text{Y}}_{1}=lnlikes;{\text{Y}}_{2}=lncomments;{\text{Y}}_{3}=lnshares$$

Y_*ij*_ [y_*i*1_ or y_*i*2_ or y_*i*3_] is the number of likes or comments or shares for each brand post *i*. Control_*i*_ is a control variable, including weekdays, posting schedule, media richness, etc.

For each blog post *i*, we formulated the basic regression model as follows:


$$\begin{array}{c} {\text{Y}}_{ij}=\alpha_{ij}+\beta_{1}contentstrategy_{ij}+\beta_{2}Affec\_response_{ij}\\ \;+\beta_{3}Inter\_response_{ij}+\beta_{4}Co\_response_{ij}\\ \;+\beta_{5}Discretionarypurchases_{ij}+\beta_{6}Brandimage_{ij}\\ \;+\beta_{7}Control_{i}+\varepsilon_{ij} \end{array}$$

To see whether discretionary purchases and brand image have a moderating effect on the relationship between brand social media response strategies and the overall level of digital customer engagement, based on previous research, we used raw scores of likes, comments and shares, calculated additional dependent variable weights and took the natural logarithm as the overall level of digital customer engagement (Coursaris et al., 2016).


lnDCE=0.5*∑(L)+0.5*∑(C)+0.5*∑(S)

$$\begin{array}{c} \text{l}nDCE_{i}=\alpha_{i}+\beta_{1}Responsestrategy_{i}+\beta_{2}Discretionarypurchases_{i}\\ \;+\beta_{3}Brandimage_{i}+\beta_{4}Responsestrategy_{i}*\\ \;Discretionarypurchases_{i}+\beta_{5}Responsestrategy_{i}*\\ \;Brandimage_{i}+\beta_{6}Control_{i}+\varepsilon_{i} \end{array}$$

## Results

According to the descriptive statistics of brand social media content strategies, the number of posts for information strategy was 397 (26.1% of the total number of posts), the number of posts for community strategy was 524 (34.5% of the total number of posts) and the number of posts for action content strategy was 598 (39.4% of the total number of posts). The majority of posts were categorized as action content, which reflects the active motivation of brands to reach consumers through social media in order to effectively motivate customers to consume. The estimation results are presented in Tables 1, 2 summarizes the findings. The impact of potential independent variables on the different levels and overall level of digital customer engagement differ significantly.

**TABLE 1:** Model results table.

	(1)	(2)	(3)

	Likes	Comments	Shares
**Independent variable** Community	–0.215**	–0.107	–0.604***
	(–2.040)	(–0.925)	(–5.302)
Action	0.177**	0.470***	0.203**
	(2.030)	(4.570)	(2.052)
Affec_response	0.249***	0.345***	0.115
	(3.370)	(4.745)	(1.397)
Inter_response	0.119	0.226***	0.086
	(1.492)	(2.613)	(0.981)
Co_response	0.171**	0.148**	0.242***
	(2.461)	(2.176)	(3.174)
Discretionary purchases	0.648***	–0.108	–1.834***
	(4.344)	(–0.653)	(–12.193)
Brand image	0.397***	0.286***	0.382***
**Control variable**	(5.046)	(3.374)	(4.283)
Post scheduling	–0.108*	–0.001	–0.017
	(–1.768)	(–0.009)	(–0.258)
Weekdays	–0.232**	–0.263***	–0.312***
	(–2.367)	(–2.722)	(–2.855)
Lenth of post	–0.000	–0.000	–0.000
	(–0.224)	(–0.391)	(–0.846)
Hashtags	0.042	0.009	0.223***
	(0.541)	(0.118)	(2.832)
Link	–0.046	0.431***	0.739***
	(–0.440)	(3.956)	(5.682)
Picture	0.244***	0.392***	0.357***
	(3.118)	(4.997)	(4.759)
Video	–0.018	–0.207***	0.120
	(–0.239)	(–2.616)	(1.400)
Number of post	0.000***	0.000***	0.000**
	(5.733)	(5.758)	(2.407)
Fans of brand	0.000***	0.000***	0.000**
	(3.757)	(3.672)	(1.990)
Posting frequency	0.041	–0.348***	–0.330***
	(1.061)	(–7.825)	(–7.688)
_cons	3.430***	3.238***	3.891***
	(13.591)	(11.653)	(13.986)
Number of obs	1,519	1,519	1,519
R-squared	19.68%	26.45%	29.32%
*F*-test	22.847	41.390	39.889
Prob > F	0.000	0.000	0.000
AIC	4806.879	4838.270	5079.557
BIC	4902.744	4934.135	5175.421

***p < 0.01, **p < 0.05, *p < 0.1.

*AIC, Akaike information criterion; BIC, Bayesian information criterion.*

**TABLE 2:** Summary of results.

Hypothesis	Positive filtering	Cognitive and affective processing	Advocacy	Overall level of digital customer engagement
**Brand social media content strategies**				
H1: Community (VS. Information)	X	X	X	n.a.
H2: Action (VS. Information)	✓	✓	✓	n.a.
**Brand social media response strategies**				
H3: Affective response	✓	✓	X	n.a.
H4: Interactive response	X	✓	X	n.a.
H5: Cohesive response	✓	✓	✓	n.a.
Brand image				
H6: Brand image	✓	✓	✓	n.a.
H7: Brand image and brand social media response strategy	n.a.	n.a.	n.a.	✓
**Discretionary purchases**				
H8: Discretionary purchases	✓	X	✓	n.a.
H9: Discretionary purchases and brand social media response strategy	n.a.	n.a.	n.a.	✓

*n.a., not applicable, because no hypothesis is made.*

### Impact of Brand Social Media Content Strategies and Response Strategies

As shown in Table 1, we conducted a hierarchical regression analysis. The three models are generally significant and reasonably explain the variance of the dependent variable. We found significant differences in the impact of brand social media content strategies on different levels of digital customer engagement. The main positive impact of action strategy (vs. information) was significant (β = 0.177, *p* < 0.05; β = 0.470, *p* < 0.01; β = 0.203, *p* < 0.05), while the information strategy can generate more likes and shares compared to the community strategy (β = –0.215, *p* < 0.05; β = –0.604, *p* < 0.01), but the effectiveness of the community strategy in comments did not differ from the information strategy (β = –0.107, *p* = 0.355). We think that due to the high commercial intent of Weibo, for example, the content presentation of the reward involved in the action strategy is directly related to consumer interests and may be more likely to generate “likes” among users and motivate them to share in an organic way. Information strategy, while favoring traditional “informing” communication, have additional brand preferences based on behavioral motivations for brand equity, and consumers are willing to accept information with high perceived brand expertise. Among the response strategies, interactive response had a non-significant main effect on likes and shares (β = 0.119, *p* = 0.136; β = 0.086, *p* = 0.327), but a positive and significant effect on comments (β = 0.226, *p* < 0.01), and affective response was significantly different from interactive response, which instead had a significant increase in the main effect on likes and comments, but not having a significant effect on shares. As suggested in H5, cohesive response does significantly predict the different levels of digital customer engagement.

### The Direct Impact of Brand Image and Discretionary Purchases

As shown in Table 1, we found differences in the impact that discretionary purchases has on different levels of digital customer engagement. At the level of positive filtering, experiential brands could generate more likes compared to material brands (β = 0.648, *p* < 0.01); while at the level of cognitive and affective processing, there was no significant difference between the two purchases types (β = –0.108, *p* = 0.514); however, at the level of advocacy, material brands had a more positive impact than experiential brands (β = –1.834, *p* < 0.01), which is an important finding that differs from previous studies. Second, in the area of brand image research, consistent with predictions, the “warmth” image was more attractive compared to the “competence” image and had a significantly higher impact on the three levels of digital customer engagement.

### Moderating Effect of Brand Image and Discretionary Purchases

**FIGURE 1:**
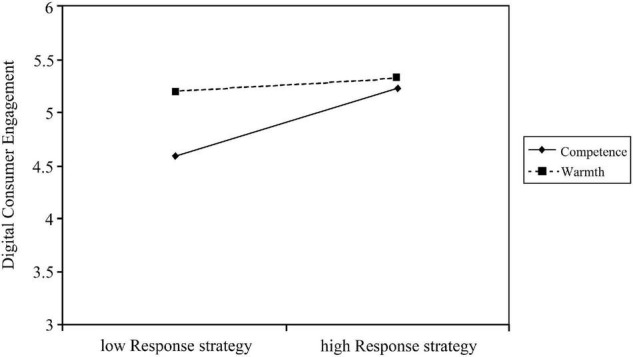
Moderating effect of brand image on the relationship between brand social media response strategy and digital customer engagement.

After controlling variables such as media richness and weekdays, consistent with our hypothesis, the mean difference between high and low response strategies was greater for the “competence” brand image. The moderating effect of “competence” brand image on digital customer engagement is more significant than that of “warmth” brand image, implying that brands with a more prominent “competence” image have a significantly higher digital customer engagement by increasing their response strategies (see Table 1). This implies that brands that highlight the “competence” image have a significant increase in digital customer engagement by advancing their response strategies. As shown in Figure 1, overall, “warmth” image has a higher level of digital customer engagement than the “competence” image, with a small increase in impact. However, when the brand image highlights the organizational competence, the more positive response strategy has a more significant impact on the digital customer engagement.

**FIGURE 2:**
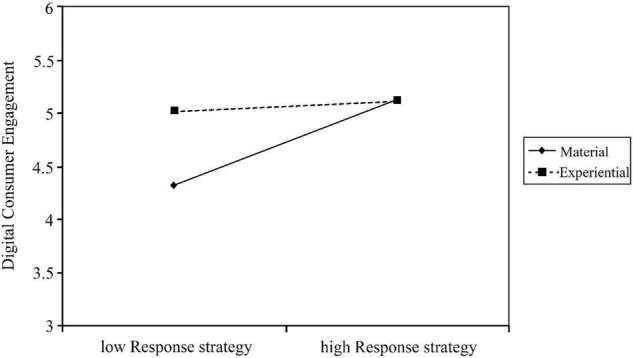
Moderating effect of discretionary purchases on the relationship between brand social media response strategy and digital customer engagement.

Next, as shown in Figure 2, in the comparison between experiential purchases and material purchases, there is a considerable increase in the slope of the fitted line for material purchases as the response strategy increases, thus showing that the higher the brand social media response strategy based on material purchases, the higher the digital customer engagement level. However, it is worth noting that brands with predominantly experiential purchases have little difference in the impact of high and low response strategies on digital customer engagement.

## Discussion

This study integrates two dimensions of content strategies of brand message and response strategies of social presence, extends the theoretical framework of brand social media strategies, and empirically tests it in a differentiated framework of brand image and discretionary purchases. To do so, we conducted a multiple linear regression analysis using data collected from Weibo. Our results extend the literature by providing novel insights into the role of DCEs within a social media context. We aim to answer the three research questions mentioned above. Regarding the first questions, this study compares the effects of three content strategies on different levels of digital customer engagement. Compared to the other two types of content strategies, action content strategy had a significant positive impact on different levels of digital customer engagement. The findings indicate that the action content strategy is the most effective in stimulating cognitive and affective processing as well as advocacy. In contrast to the previous findings, community content strategy was less effective in stimulating digital customer engagement due to higher interactive requirements, where information content strategy was more effective than community content strategies in positively filtering and advocacy. Regarding the second question, brand social media response strategies have a mixed impact on digital customer engagement. Cohesive response strategies are the most effective way to promote digital customer engagement. A key advantage of a cohesive response strategy is that it focuses on highlighting brand affinity and showcasing the human side of the brand to create familiarity and intimacy. Regarding the third question, in the brand image dimension, “warmth” is associated with a higher level of digital customer engagement compared to “competence.” In the dimension of discretionary purchases, we find no significant differences between material purchases and experiential purchases on the cognitive and affective processing dimension of digital customer engagement. In addition, we find that the level of brand social media response strategy has a greater impact on digital customer engagement in terms of “competency” brand image and material purchases, the higher the response level, the higher the digital customer engagement level.

### Theoretical Implication

This study makes some initial contributions to the customer engagement and brand literature, and from it we draw three main theoretical insights. First of all, this study focuses on the effectiveness of two categories of brand social media strategies, which are content strategy and response strategy, in terms of digital customer engagement. The study shows that in terms of content strategies, action content strategy has the most significant impact on digital customer engagement, with the largest share at the level of facilitating shares. Rewarding and gamified content strategies play a crucial role in triggering digital customer engagement. Brands should proactively incentivize online users, with incentives such as monetary incentives and relationship incentives, which are expected to achieve higher level of digital customer engagement. Information strategy effectively influences the likes and shares levels of digital customer engagement, so brands can focus on presenting information about product or service quality aspects and corresponding detailed experiences or descriptions that reflect brand competence and customer preferences. Next, contrary to our hypotheses, the community strategy did not obtain higher level of digital customer engagement than the information strategy, which may be due to the fact that community requires the establishment of deeper level of interaction as part of self-expression. In addition, we extend social presence theory to social media contexts and have distinguished three types of response strategies based on social presence theory, each of which differentially influenced different levels of digital customer engagement. The results show that this study does not find that all response strategies significantly affect digital customer engagement. The impact of response strategies on digital customer engagement is not homogeneous and requires different corresponding strategies based on different goals, which ultimately lead to different levels of digital customer engagement. For example, affective response strategy tends to exhibit more positive cognitive and affective processing behaviors, while cohesive response strategy is more influential in increasing the number of advocacy. Lastly, most of the previous studies take single-focus brands into consideration and do not extend it to be the differentiated impact on digital customer engagement between different brand types. Therefore, we illustrate that digital customer engagement is influenced by brand image and discretionary purchases differentiation through two comparative studies of brand image and discretionary purchases, and the findings reveal the interaction of brand social media response strategies with brand image and discretionary purchases and demonstrate the extent to which they influence digital customer engagement, further enriching the existing insights.

### Management Implication

This study has several enlightening insights for managers or marketers who use social media. First, social media offers greater flexibility in terms of usefulness, relevance, timeliness, and customization of information. This study encourages managers to consider brand social media strategy as an important element of their brand strategies. We use the most common engagement metrics to look at digital customer engagement and link brand social media strategies to these metrics and further stimulate user engagement, trust, and relationship development. We recommend that brands carefully measure and examine digital customer engagement behaviors, considering consumer behaviors in general, including the behaviors across different platforms, devices, and digital touchpoints. Second, from a practical perspective, managers are advised to carefully select, design, and promote brand-related messages on social media pages, including goals related to the marketing purpose, multi-stakeholder impact, and a high degree of brand personality/cultural identity engagement. In addition, managers need to enrich and leverage the functional interactive features of social networks to improve digital customer engagement. However, many brands rarely respond to user comments, and social media’s conversational features are underutilized. Therefore, brands can demonstrate brand personality and sincerity through conversations to promote mutual understanding between brands and consumers. Finally, the focus dimension of customer engagement may be different for different brand image and discretionary purchases. This study focuses on the impact of differentiated attributes of brands on digital customer engagement, which helps to strengthen authentic and effective marketing communication, provide product and brand matching differentiated service operations, and strengthen coordination capabilities with partner brands to continuously improve the level of digital customer engagement.

### Limitation and Future Research

This study exists limitations. First, this study explores brand social media strategies and digital customer engagement from a micro perspective. For future research, we suggest exploring other perspectives, e.g., the socio-technical phenomenon based on a socio-technical perspective to explore the integration of digital engagement behavior with digital technology. Second, the data used in this study is limited to the official social media pages of each brand. We suggest further exploring the factors that influence digital customer engagement, e.g., leader self-disclosure, opinion leaders, and group opinions. Future research should strive to extend social media data, such as whether opinion leader engagement leads to higher digital customer engagement or whether it affects the conversion of digital customer engagement validity. Third, digital customer engagement can vary by media context. There is only one social platform selected for this study, resulting in the lack of information on other social platforms, which implies that consumers who use social platforms such as Tiktok or WeChat or other platforms simultaneously may have self-selection bias. Cross-platform domain research can help to fully take in the relationship between social media and digital customer engagement. Fourth, we recommend that researchers further investigate, test, and validate more granular aspects of digital customer engagement. Future researchers can delve deeper into digital customer engagement types and how engagement types are linked to downstream outcomes (e.g., purchases rates or brand value). Finally, there are limitations to a single source of brand expressiveness, and many brands optimize their brand resourcing and integration capabilities through brand association mechanisms. The future researchers can focus on exploring whether the differentiated or consistent choice of brand association type facilitates the generation and validity of customer engagement.

## Data Availability Statement

The raw data supporting the conclusions of this article will be made available by the authors, without undue reservation.

## Author Contributions

YL and DW contributed to the conception and design of the study. YL, XL, and MW performed data collection, collation, interpretation, and the statistical analysis. YL wrote the first draft of the manuscript. DW was leading the further elaborations and revisions. All authors contributed to the manuscript revision, read, and approved the submitted version.

## Conflict of Interest

The authors declare that the research was conducted in the absence of any commercial or financial relationships that could be construed as a potential conflict of interest.

## Publisher’s Note

All claims expressed in this article are solely those of the authors and do not necessarily represent those of their affiliated organizations, or those of the publisher, the editors and the reviewers. Any product that may be evaluated in this article, or claim that may be made by its manufacturer, is not guaranteed or endorsed by the publisher.
